# Emerging Roles of Platelets in Allergic Asthma

**DOI:** 10.3389/fimmu.2022.846055

**Published:** 2022-04-01

**Authors:** Ming Yue, Mengjiao Hu, Fangda Fu, Hongfeng Ruan, Chengliang Wu

**Affiliations:** ^1^ Department of Physiology, College of Basic Medical Sciences, Zhejiang Chinese Medical University, Hangzhou, China; ^2^ Department of Immunology and Microbiology, College of Basic Medical Sciences, Zhejiang Chinese Medical University, Hangzhou, China; ^3^ Institute of Orthopaedics and Traumatology, The First Affiliated Hospital of Zhejiang Chinese Medical University, Hangzhou, China

**Keywords:** platelets, allergic asthma, dendritic cells, eosinophils, platelet-derived mediators, antiplatelet strategies

## Abstract

Allergic asthma is a complex chronic inflammatory disease of the airways, driven by Th2 immune responses and characterized by eosinophilic pulmonary inflammation, airway hyperresponsiveness, excessive mucus production, and airway remodeling. Overwhelming evidence from studies in animal models and allergic asthmatic patients suggests that platelets are aberrantly activated and recruited to the lungs. It has been established that platelets can interact with other immune cells and secrete various biochemical mediators to promote allergic sensitization and airway inflammatory response, and platelet deficiency may alleviate the pathological features and symptoms of allergic asthma. However, the comprehensive roles of platelets in allergic asthma have not been fully clarified, leaving attempts to treat allergic asthma with antiplatelet agents questionable. In this review, we summarize the role of platelet activation and pulmonary accumulation in allergic asthma; emphasis is placed on the different interactions between platelets with crucial immune cell types and the contribution of platelet-derived mediators in this context. Furthermore, clinical antiplatelet approaches to treat allergic asthma are discussed. This review provides a clearer understanding of the roles of platelets in the pathogenesis of allergic asthma and could be informative in the development of novel strategies for the treatment of allergic asthma.

## Introduction

Asthma is a chronic inflammatory disease of the airways, affecting more than 300 million people worldwide (1). Allergic asthma is widely acknowledged as the most common asthma phenotype, characterized by type 2 inflammation, involving a variety of cells, especially dendritic cells (DCs), T helper 2 (Th2) cells, B cells, eosinophils, mast cells, and basophils, as well as immunoglobulin E (IgE) and type 2 cytokines such as IL-4, IL-5, IL-13, and inflammatory chemical mediators (2, 3). Upon allergen exposure, DCs are activated and mature in the presence of epithelium-derived cytokines, acquiring the ability of inhaled allergen uptake followed by allergen presentation to naive CD4^+^ Th cells, stimulating them to differentiate into Th2 cells that secrete type 2 cytokines. Importantly, B cells preferentially produce IgE in the presence of IL-4 and IL-13. Mast cells and basophils are targeted by IgE and undergo degranulation after crosslinking of IgE by re-exposed allergens. Meanwhile, eosinophils are recruited into the lung and release many inflammatory mediators (3). In susceptible patients, inflammatory mediators can cause airway hyperresponsiveness, mucus overproduction, and airway remodeling, which lead to repeated episodes of wheezing, shortness of breath, coughing, and chest tightness (4, 5). The current therapeutic strategy for allergic asthma encompasses bronchodilators [short- and long-acting β2-adrenoceptor agonists (SABAs and LABAs)], glucocorticoids, environmental controls, allergen immunotherapy, and biological agents targeting the Th2 pathway (6, 7). However, these interventions are reportedly ineffective in alleviating the aforementioned symptoms in some patients with allergic asthma (8, 9). Therefore, it is urgent to further elucidate the cellular and molecular mechanism of allergic asthma to facilitate the development of novel therapies.

**Figure 1:**
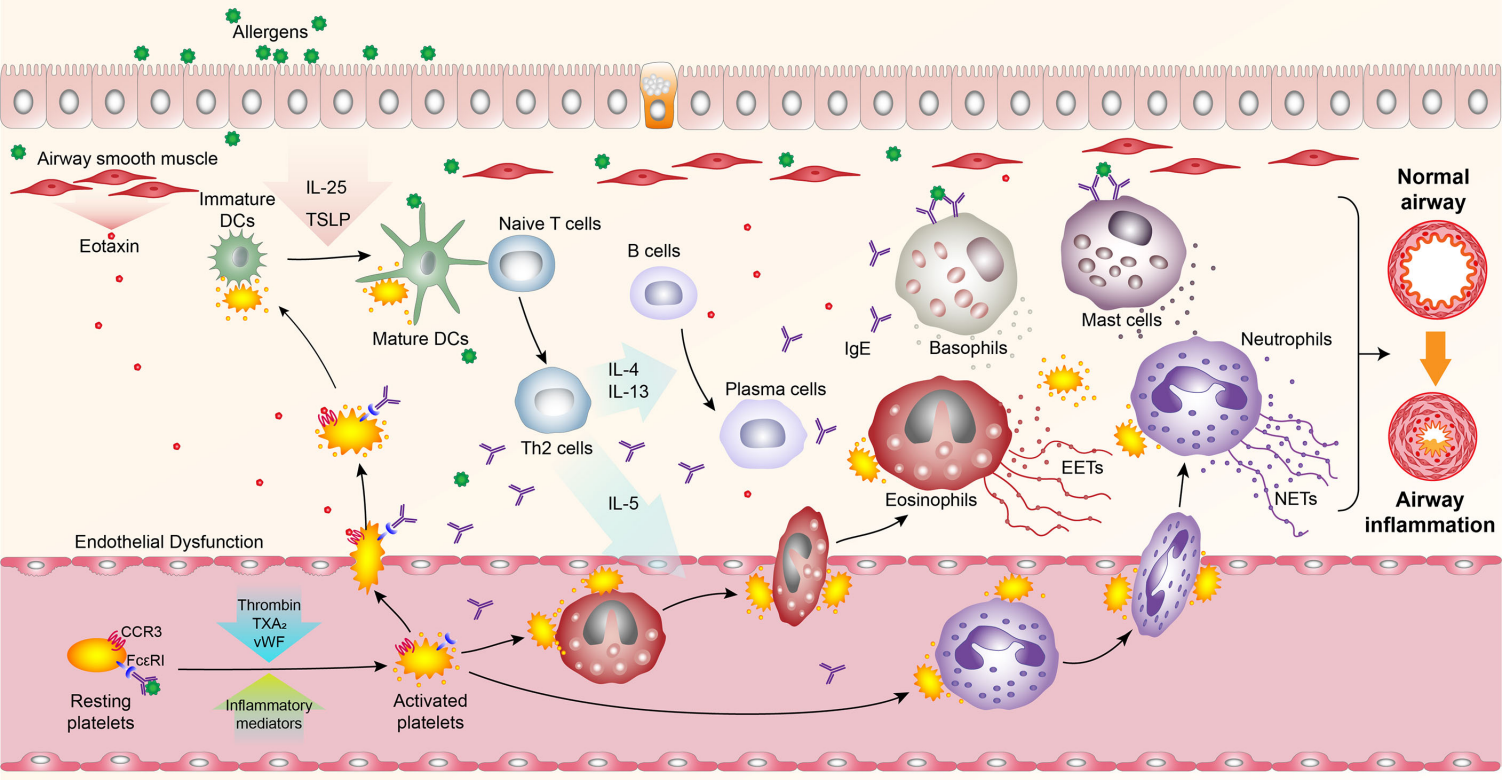
Overview of platelet activation, pulmonary recruitment, interactions with leukocytes, and secretion in allergic asthma. Upon allergen exposure, platelets are activated, and recruited into the lungs in an FcϵRI- and CCR3- dependent manner, where they bind to dendritic cells (DCs) and secrete mediators to promote the maturation of DCs. Activated circulating platelets also interact with eosinophils and neutrophils to induce their recruitment into the lungs and promote the formation of eosinophil extracellular traps (EETs) and neutrophil extracellular traps (NETs). Meanwhile, platelet-derived mediators can induce airway inflammatory responses.

Platelets are small anucleate cellular fragments derived from megakaryocytes, and one trillion platelets circulate in the adult blood (10, 11). Apart from their critical roles in hemostasis and thrombosis, platelets are also considered immune cells participated in various immune-related processes (12). An increasing body of evidence suggests that in patients with allergic asthma, platelet-specific derived mediators such as platelet factor 4 (PF4) and beta-thromboglobulin (β-TG) increase in the serum, indicating platelet activation (13–20). A functional validation study further showed that depletion of platelets could significantly relieve allergic asthma symptoms, substantiating the importance of platelets in allergic asthma (21). Interestingly, numerous studies have reported that platelets are recruited and localized in lung tissues, participating in pulmonary inflammation reactions by interaction with DCs, eosinophils, and neutrophils to alter their functions (22–26). Meanwhile, other experiments corroborated that platelets could release a variety of mediators (27), such as growth factors (28–34), ATP (35), histamine (36), serotonin (37), IL-33 (38), platelet-activating factor (PAF) (39, 40), thromboxane A_2_ (TXA_2_) (41) to exacerbate airway inflammation (**Figure 1**).

In this review, we briefly introduced the role of platelet activation and intrapulmonary recruitment of platelets in allergic asthma. Subsequently, we reviewed the latest findings on the interactions between platelets and vital immune cells, including dendritic cells, eosinophils, and neutrophils, as well as platelet-derived mediators, and their roles in the pathophysiology of allergic asthma. The current antiplatelet therapies for allergic asthma were listed and analyzed.

## Activation and Pulmonary Recruitment of Platelet in Allergic Asthma

### Platelet Activation in Allergic Asthma

It is well-established that under physiological conditions, platelets circulate in the bloodstream exhibiting a discoid shape in the resting state. Three types of secretory granules in platelets carry large amounts of active substances, including α-granules, dense granules, and lysosomes (42). Upon platelet activation, these granules undergo exocytosis and release contained substances, including platelet-specific derived mediators such as PF4, β-TG, soluble P-selectin, soluble CD40 ligand (sCD40L), and thrombospondin-1 (TSP-1). During this process, P-selectin translocates from α-granules to the platelet surface, and integrin αIIbβ3 (also known as glycoprotein IIbIIIa) undergoes conformational changes, obtaining a high affinity for its ligand (43–45). These findings suggest that platelet activation can be monitored by detecting platelet-specific derived mediators and platelet-specific surface markers by enzyme-linked immunosorbent assay (ELISA) and flow cytometry, respectively (43). The detailed information on platelet activation has been discussed in another review (43).

In 1981, a study reported that plasma PF4 was increased in patients with allergic asthma, providing the first evidence of the relationship between platelet activation and allergic asthma (13). There is ample evidence suggesting that platelet-specific products, including PF4, β-TG, and platelet microparticles, are elevated in allergic asthma subjects, confirming the presence of platelet activation in allergic asthma (14–20). Nowadays, a more detailed mechanism of platelet activation in allergic asthma has been documented, involving three different pathways for triggering platelet activation. **1)** Inflammation induces platelet activation. Inflammatory reaction causes endothelial dysfunction, leading to increased production of thromboxane A_2_ (TXA_2_), von Willebrand factor (vWF), and thrombin, which are potent platelet agonists. Many inflammatory mediators such as proinflammatory cytokines or PAF also activate platelets (46–48). Additionally, CD40L-positive T cells in immune responses can activate platelets by interacting with CD40 on platelets (49, 50). **2)** IgE generated during allergic reactions mediates platelet activation. Importantly, platelets express high- and low-affinity IgE receptors (FcϵRI and FcϵRII) (51–53). Moreover, current evidence suggests that in allergic asthmatic patients, the binding of IgE to platelets leads to their direct activation after contact with the corresponding allergens (50). **3)** Different allergens may generate different mediators to activate platelets. In Candida albicans-induced airway infection, the fungi peptide toxin candidalysin was found to activate platelets by binding to GPIbα on platelets, resulting in the secretion of Wnt antagonist Dickkopf-1 (Dkk-1), which then drives allergic reactions (54).

### Intrapulmonary Recruitment of Platelets in Allergic Asthma

Previous investigations on platelets have mainly focused on the biological processes in the vasculature. Accumulating evidence suggests that platelets migrate out of vessels and localize underneath the airways in allergic asthma subjects, enabling them to participate in tissue inflammation directly. Moreover, platelets form aggregates with leukocytes in the circulating blood of patients with allergic asthma, which contribute to the latter’s pulmonary recruitment (25). Surprisingly, further evidence showed that platelet migration precedes leukocyte trafficking into lung tissue and a high proportion of platelets are not adjacent to leukocytes, suggesting that platelets are not just accessory factors for leukocytes in tissue inflammation (22, 23).

The recruitment of platelets into the lung is largely dependent on the IgE receptor FcϵRI given that platelet penetration into the lungs and platelet chemotaxis toward ovalbumin (OVA) are severely impaired by *FcϵRI* deficiency (22). Moreover, platelets express chemokine receptors CCR3, CCR4, and CXCR4, which have been reported to control myeloid-cell migration during allergic inflammation. As a result, platelets undergo migration in response to the ligands of CCR3, CCR4, and CXCR4 (23). Paradoxically, in an allergic asthmatic mouse model, inhibition of CCR3 and CCR4 but not CXCR4 reduced platelet migration into the lungs. Besides, the expression of eotaxin, the chemokine ligand for CCR3, was increased in the airway smooth muscle of allergic asthmatic patients, which further promoted intrapulmonary recruitment of platelets. These findings suggest that the chemokine receptor CCR3 is essential in pulmonary platelet recruitment. However, it is noteworthy that CXCR4 inhibition significantly reduces the recruitment of leukocytes to the lungs, suggesting that the recruitment of platelet and leukocyte involves different pathways (23).

## Multiple Roles of Platelets in Allergic Asthma

### Roles of Platelet-DC Interactions in Allergic Sensitization

**Figure 2:**
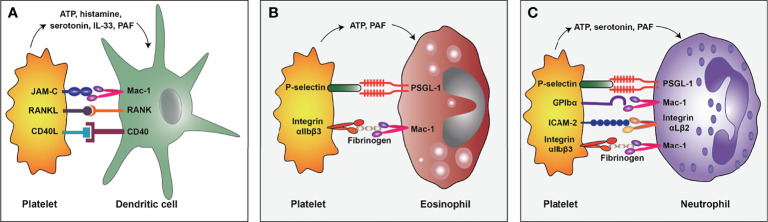
The interaction mode of platelet and dendritic cell **(A)**, eosinophil **(B)**, and neutrophil **(C)** in allergic asthma. PSGL-1, P-selectin GP ligand-1; ICAM-2, Intercellular adhesion molecule-2; PAF, platelet-activating factor.

DCs have been documented to play a critical role in allergen sensitization (55). In this regard, DCs migrate to the secondary lymphatic organs after the uptake of inhaled allergens, presenting the processed antigens to naive CD4^+^ Th cells and promoting Th2 cell differentiation (56). A recent study documented that platelets and DCs were co-localized around the airway walls in allergen-sensitized mice, and transient platelet depletion could inhibit the pulmonary recruitment of DCs during the sensitization process (57). In addition, activated platelets can aggregate to thymic stromal lymphopoietin (TSLP)-stimulated DCs to induce the maturation of DCs and the production of CCL17 (a Th2 cell-attracting chemokine) (24). These studies demonstrated that interactions between platelets and DCs are crucial for allergen sensitization. Furthermore, Mac-1 (also known as integrin CD11b/CD18) on DCs and junctional adhesion molecules (JAM)-C on platelets, RANK on DCs and RANKL on platelets, as well as CD40 on DCs and CD40L on platelets, may contribute to these interactions (24, 58, 59) (**Figure 2A**). Interestingly, platelet-derived mediators such as ATP (35), histamine (60), and serotonin (61) have also been identified to promote the maturation and Th2 priming capacity of DCs.

### Roles of Platelet-Eosinophil Interactions in Allergic Asthma

Eosinophilic infiltration of the airway has been established as a hallmark of allergic asthma. Mounting evidence indicates that eosinophils migrate to the bronchial tissue during the late-phase asthmatic response (62, 63) and secrete large amounts of toxic proteins and proinflammatory mediators, leading to bronchoconstriction, airway hyperresponsiveness, and mucus overproduction (3). Importantly, eosinophil recruitment from the circulation requires their activation (64, 65), which has been reported to be positively associated with platelet activation in asthmatic airways (66, 67). Moreover, it has been shown that platelets can interact directly with eosinophils to mediate pulmonary eosinophil recruitment in a mouse model of OVA-induced asthma (25).

Mechanistic studies have demonstrated that platelet-eosinophil interactions involve platelet P-selectin and integrin αIIbβ3 (**Figure 2B**). P-selectin is an adhesion molecule, and its counter-receptor is P-selectin GP ligand-1 (PSGL-1) (68). It has been shown that platelets lacking P-selectin are unable to mediate eosinophil recruitment (25). Similarly, blockade by antibodies against P-selectin can reduce the binding and clustering abilities of eosinophils on the endothelium of allergic asthma patients (62). Further studies indicated that platelet P-selectin is required for the activation of eosinophil β1 integrin (69), which pairs with α4-integrin and then binds to the vascular cell adhesion molecule-1 (VCAM-1) of endothelial cells, mediating the rolling, adhesion, and migration of eosinophils on the endothelium (70). Current evidence suggests that integrin αIIbβ3 is a major membrane protein on platelets and is crucial for platelet aggregation (45, 71). Besides, a study documented that the inhibition of integrin αIIbβ3 by tirofiban could significantly reduce the platelet-eosinophil aggregates and pulmonary eosinophil infiltration in allergic airways and reduce airway hyperresponsiveness and inflammation (72). Mechanistic studies further indicated that Mac-1 on eosinophils could interact with integrin αIIbβ3 through a bridge molecule such as fibrinogen and sCD40L (73, 74).

It has been established that eosinophil extracellular traps (EETs), composed of DNA and major basic protein (MBP), are almost universally formed in the allergic asthmatic airways and associated with the severity of asthma (75). There is overwhelming evidence that EETs can increase proinflammatory cytokine production, airway epithelial permeability, goblet-cell hyperplasia, mucus production, and pulmonary inflammation during allergic asthma (75, 76). A negative correlation has also been found between the levels of EETs and pulmonary ventilatory function in asthmatic patients (76). Moreover, a mechanistic investigation revealed that EETs could activate pulmonary neuroendocrine cells (PNECs) and amplify allergic immune responses *via* neuropeptides and neurotransmitters (75). In recent years, platelet-eosinophil interactions have been shown to induce EETs formation to enhance thrombus stability in thrombosis (77). Accordingly, it is reasonable to speculate that these interactions may also be involved in the formation of EETs during allergic asthma.

### Potential Roles of Platelet-Neutrophil Interactions in Allergic Asthma

Although allergic asthma is characterized by eosinophilic airway inflammation, neutrophils are also reportedly activated and critical in disease pathogenesis (78), especially in infection-related allergic asthma (79). Importantly, chemotaxis, phagocytic activity, and ROS production of neutrophils can be induced by allergen challenge in allergic asthma patients (80). Further evidence demonstrated that pulmonary recruitment of neutrophils is dependent on CXCR2 (the receptor for CXCL1, CXCL2, and CXCL5) during allergy challenges (78, 81). Importantly, blocking CXCR2 was found to inhibit lung recruitment of neutrophils and reduce allergic sensitization and airway inflammation (81). Beyond that, neutrophils have been shown to function as antigen-presenting cells (APCs) to activate T cells. Once the allergen is ingested, neutrophils present antigens to allergen-specific T cells to activate them and display a Th2-phenotype, producing a large amount of IL-4, IL-5, and IL-13 (82).

Interestingly, neutrophils recruited to the lungs function as APCs to present allergens to T cells and release neutrophil elastase (NE) and NETs to promote airway inflammation. As a major proteinase in primary granules in neutrophils (83), NE is released into the lungs after neutrophilic recruitment (84), inducing airway remodeling by upregulating the expression of chemokines CCL24 and IL-33 in epithelial cells (84), increasing mucus production and destroying ciliary motility (83). As expected, inhibition of NE could alleviate asthmatic airway inflammation (84). In addition, NETs have been documented in the airways of allergic asthma patients (85). Besides, a study found that exposure to a low dose of lipopolysaccharides (LPS) could upregulate CXCR4 expression on neutrophils recruited to the lungs, triggering the release of NETs by these neutrophils in mouse models of allergic asthma (79). It is widely believed that exacerbation of allergic asthma largely depends on the formation of NETs and NET-associated dsDNA. Intriguingly, NET-associated dsDNA contributes to pulmonary recruitment of monocyte-derived DCs (86), producing proinflammatory chemokines and presenting allergens to T cells (87). Moreover, injection of exogenous dsDNA has been reported to directly stimulate naive CD4^+^ Th cells to induce Th2 cell differentiation and activation and exhibit exacerbation features of type 2 inflammation and asthma pathology (86).

Platelets have been shown to interact with neutrophils and promote their activation and NETs formation in a wide range of inflammatory conditions (88). Despite the lack of relevant studies on platelet-neutrophil interactions in allergic asthma, we hypothesize that platelet-neutrophil interactions may exist and participate in the pathogenesis of allergic asthma given that platelet-leukocyte aggregates have been found in allergic asthmatic patients (89) and neutrophils are the most abundant leukocytes in the blood (78). The molecular mechanisms underlying platelet-neutrophil interactions have been discussed in detail in other reviews, involving several surface proteins on platelets and neutrophils, including P-selectin/PSGL-1, GPIbα/Mac-1, integrin αIIbβ3/Mac-1, intercellular adhesion molecule-2 (ICAM-2)/integrin αLβ2, *etc* (26, 88) (**Figure 2C**). In the context of allergic asthma, some platelet-derived mediators, such as ATP (35), serotonin (37), and PAF (39), can promote neutrophil activity. Substantial evidence suggests that ATP encourages neutrophil activation (90), pulmonary recruitment (91), and NETs formation (92). Besides, serotonin induces neutrophil degranulation (93) and recruitment to the inflammation sites (61), while PAF induces pulmonary recruitment of neutrophils in asthmatic patients (94) and plays a crucial role in neutrophil adhesion to platelets (95). Besides, platelets express pattern recognition receptors (PRRs), enabling them to recognize microbial structures during infection (96). The platelet toll-like receptor 4 (TLR4), belonging to the PRR family, is necessary for NETs formation for clearance of bacteria during sepsis (97). Accordingly, as a receptor for LPS (98), platelet TLR4 may also contribute to LPS-triggered NETs formation in infection-related allergic asthma.

### Roles of Platelet-Derived Mediators in Allergic Asthma

**Figure 3:**
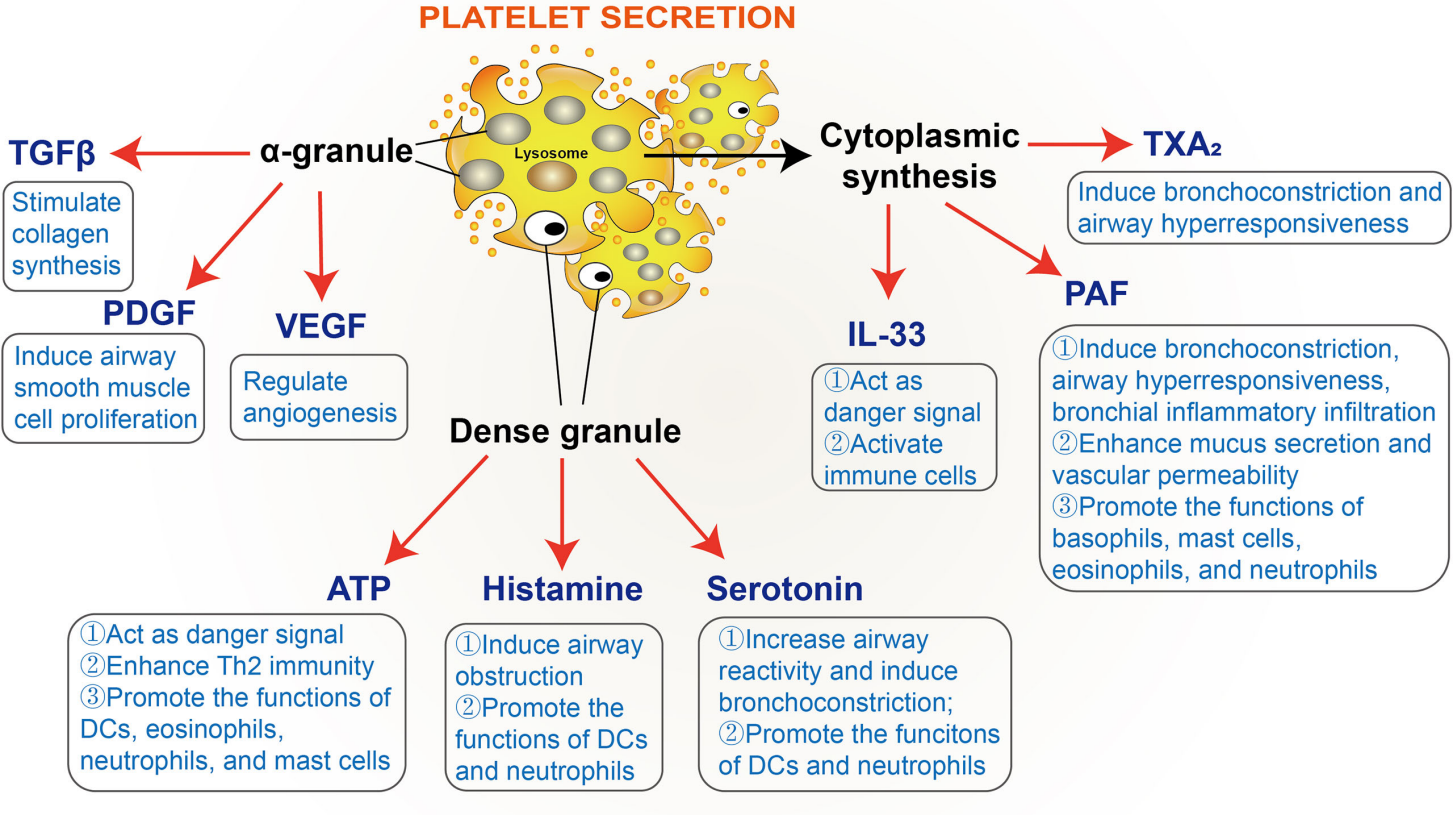
Platelet-derived mediators involved in allergic asthma. TGFβ, transforming growth factor β; PDGF, platelet-derived growth factor; VEGF, vascular endothelial growth factor; PAF, platelet-activating factor; TXA_2_, thromboxane A_2_.

Current evidence suggests that mediators secreted by platelets can regulate platelet function. As mentioned above, it has been established that three types of granules containing large amounts of biochemical molecules are present in platelets: α-granules, dense granules, and lysosomes. α-granules contain a wide variety of molecules, such as adhesive glycoproteins, coagulation factors, growth factors, cytokines, *etc*; dense granules contain several types of small molecules, including amines, cations, nucleotides, and polyphosphates; lysosomes contain various enzymes like many other cell types. Beyond that, platelets also synthesize multiple molecules upon activation, such as interleukin, PAF, and TXA_2_ (27, 42). The secretion of platelet granules is mediated by Soluble NSF Attachment Protein Receptor (SNARE) proteins and SNARE regulatory proteins (99). Moreover, Munc13 family proteins are well-recognized as essential SNARE regulatory proteins, and Munc13-4 is the only Munc13 family member expressed in platelets. *Munc13-4* deficiency has been shown to inhibit dense granule secretion from platelets and severely impair α-granule and lysosomal secretion (100). Likewise, in an OVA-induced mouse allergic asthmatic model, the platelet-specific *Munc13-4* knockout mice exhibited reduced airway hyperresponsiveness and eosinophilic inflammation. Taken together, these findings demonstrate that platelet granule secretion is vital for the progression of allergic asthma (101). Moreover, a large number of mediators involved in allergic asthma have been documented in platelet granules, such as ATP, histamine, serotonin, transforming growth factor β (TGFβ), platelet-derived growth factor (PDGF), and vascular endothelial growth factor (VEGF), *etc* (28–37). Several molecules, such as IL-33, PAF, and TXA_2_, synthesized by platelets, have also been found to promote allergic reactions (27, 38–41) (**Figure 3**).

#### Growth Factors and Airway Remodeling

Airway remodeling is a well-established pathological feature of allergic asthma (102), characterized by epithelial injury, goblet cell hyperplasia, subepithelial layer thickening, airway smooth muscle hyperplasia, and angiogenesis (103). Platelets have been documented to play pivotal roles in airway remodeling in allergic asthma, given that platelet depletion could suppress epithelial and smooth muscle thickening and deposition of reticular fibers in the extracellular matrix (ECM) in a mouse model of allergic asthma (104). Indeed, the α-granules of platelets contain various growth factors (11), some of which are significantly increased in allergic asthmatic patients and play an important role in airway remodeling, such as TGFβ (28), PDGF (29), and VEGF (30–34).

Current evidence suggests that TGFβ stimulates collagen synthesis (105), PDGF induces airway smooth muscle cell proliferation (106, 107), and VEGF is a vital regulator of angiogenesis (108), strongly associated with the expression of MMP-9 (109), playing a central role in matrix degradation in injury and contributing to tissue remodeling (110). These growth factors have become promising pharmaceutical targets in the treatment of asthma. For instance, inhibitors of PDGF receptors, imatinib, nilotinib, and masitinib, have been studied to treat asthma. In this regard, imatinib and nilotinib have been found to reduce leukocyte recruitment, collagen deposition, and smooth muscle layer thickening, and masitinib administration reduced eosinophilia and the total protein in bronchoalveolar lavage fluid (29). In a randomized, double-blind, placebo-controlled phase 2 study (which initially included 62 poorly controlled patients with severe asthma and 50 finished the study), an oral dose of 400 mg/day imatinib has been associated with decreased airway hyperresponsiveness, mast cell counts, and tryptase release. A 16-week randomized placebo-controlled study, including 44 patients with severe corticosteroid-dependent asthma, showed that masitinib (with doses ranging 3, 4.5, and 6 mg/kg/day) could improve the Asthma Control Questionnaire score (29). Meanwhile, anti-VEGF treatment could significantly reduce the thickness of the epithelium, subepithelial muscle, basement membrane, and the number of goblet cells and mast cells in an OVA-induced mouse allergic asthmatic model (111).

#### Nucleotides

It is widely acknowledged that many nucleotides (about 436 mM ATP and 653 mM ADP) are packaged in platelet dense granules (27, 112). Studies have shown that ATP can bind to a ligand-gated ion channel receptor P2X1R on platelets and causes calcium to flow into the platelets, promoting platelet activation. Nonetheless, ATP alone cannot induce platelet aggregation (113). ADP is a very important autocrine agonist for platelet aggregation and thrombosis, and its receptors on platelets are P2Y1R and P2Y12R. Clinically, P2Y12R antagonists are commonly used antiplatelet therapies against cardiovascular diseases.

Although ATP is not a potent agonist of platelet activation, it is considered a dangerous signal in the immune system. Elevated ATP levels have been documented in bronchoalveolar lavage fluid from patients with allergic asthma and murine asthma model (35). ATP can induce DC maturation and migration (35), eosinophil migration, adhesion, and degranulation (114, 115), as well as mast cells degranulation (116). Indeed, suppressing airway ATP levels or inhibiting purinergic P2 receptors may reduce airway inflammation (35). Studies have shown that ATP exerts its effects through the purinergic P2 receptors, which can be subdivided into two families: the ligand-gated ion channels P2XR and the G-protein-coupled P2YR. Over the years, P2X7R and P2Y2R have been extensively studied in allergic asthma. The expression of P2X7R is up-regulated in bronchoalveolar lavage fluid of allergic asthmatic patients and mice. Importantly, the inhibition or absence of P2X7R could reduce the capacity of DCs to induce Th2 immunity and airway inflammation of allergic asthmatic mice (117). Furthermore, it has been shown that children with attenuated P2X7R function have lower susceptibility to asthma (118), and P2Y2R expression on human DCs and eosinophils was upregulated in an allergic asthma model (119). *P2Y2R* deficiency impairs the recruitment of DCs and eosinophils and attenuates allergic airway inflammation (119, 120). Additionally, P2Y2R has been reported to be a regulator of the membrane and soluble form VCAM-1, vital for the adhesion and filtration of eosinophils (120). These findings substantiate that P2X7R and P2Y2R are key receptors for ATP-mediated allergic responses.

#### Histamine

Histamine is one of the earliest identified mediators of allergy, which can be secreted from platelet dense granules (27, 121, 122). Histamine contributes to airway obstruction by affecting smooth muscle contraction, bronchial secretion, and airway mucosal edema (36). Moreover, histamine can promote DC differentiation, antigen uptake, presentation, and migration, as well as T cell polarization (60, 123). It is well-recognized that histamine has four receptors (H1R, H2R, H3R, and H4R), among which H1R and H4R have been associated with allergic asthma (124) and mediate T cell chemotaxis to histamine. In this respect, a study demonstrated that *H1R* knockout mice failed to develop lung inflammation and local Th2 responses following allergen challenge, and *H1R*-deficient T cells could not migrate to the antigen exposure site (125). Moreover, the H1R antagonist terfenadine was found to reverse decreased FEV_1_ induced by allergen inhalation to a certain extent (36). The role of H4R in asthma remains controversial. It has been reported that mice lacking *H4R* or treated with H4R antagonists exhibit decreased allergic airway inflammation and Th2 responses (126). A contrasting study documented that activation of H4R prior to allergen challenge could mitigate airway hyperreactivity and inflammation. Stimulation of H4R could lead to the enrichment of FoxP3^+^ T cells to suppress the proliferation of autologous T cells (127).

#### Serotonin

Platelets are well-established as the primary source of serotonin in allergic asthma (37). Peripheral serotonin is first synthesized by intestinal enterochromaffin cells through tryptophan hydroxylase 1 (TPH1) and then released into blood plasma for further uptake by platelets through serotonin transporter (SERT) and stored in dense granules (128). Increased serotonin levels have been documented in asthma, positively correlating with the clinical severity rating (129). Moreover, elevated serotonin increases airway reactivity and causes bronchoconstriction (130, 131). Interestingly, it has been shown that loss-of-function of *TPH1* could reduce type 2 cytokine production, pulmonary leukocyte recruitment, and airway hyperresponsiveness in OVA-sensitized and -challenged mice (37). Consistently, tianeptine, a serotonin uptake-enhancing drug, has been reported to decrease the clinical severity rating and increase pulmonary ventilatory function (132–134). Furthermore, the transfusion of *TPH1*-deficient platelets into wild-type recipient mice (with lethal irradiation to destroy bone marrow) in the OVA-induced allergic asthmatic model has been shown to reduce allergic airway inflammation (37). Interestingly, serotonin has a substantial impact on the functions of DCs. Serotonin induces the migration of DCs to mediastinal lymph nodes and modulates chemokine release of DCs, inhibiting the production of the potent Th1 chemoattractant IP-10/CXCL10 in mature DCs, and promoting Th2-type chemokine macrophage-derived chemokine (MDC/CCL22) in both immature and mature DCs (135). Consistently, *TPH1*-deficient DCs exhibit distorted maturation, decreased cytokine production, and reduced Th2 priming capacity (37).

#### IL-33

In recent years, IL-33 has been identified as a member of the IL-1 family (136). Increased expression of IL-33 documented in bronchial biopsies of asthmatic patients has been found to correlate with clinical asthma severity (137, 138). Studies have shown that platelets constitutively express IL-33 protein, and platelet depletion significantly attenuates IL-33 dependent allergic airway inflammation (38). IL-33 in allergic asthma act as an ‘alarm’ alerting the immune system after endothelial or epithelial cell damage (139). IL-33 also activates many immune cells, such as DCs, basophils, mast cells, innate lymphoid cells (ILCs), and Th2 cells (140–142). In addition, single nucleotide polymorphisms (SNPs) in IL-33 have been associated with eosinophil counts, which is related to the pathogenesis of allergic asthma (143).

#### PAF

PAF is an important proinflammatory mediator significantly elevated in the blood of patients with allergic asthma. Overwhelming evidence suggests many cells release PAF during allergic asthma, including platelets (27, 39, 122, 144). It has been shown that PAF inhalation induces bronchoconstriction, airway hyperresponsiveness, bronchial inflammatory infiltration and enhances mucus secretion and vascular permeability. Besides, PAF can stimulate histamine release from basophils and mast cells and activate eosinophils (39, 40). Studies have shown that the PAF antagonist Y-Y-24180 could suppress antigen-induced airway hyperresponsiveness and eosinophil infiltration (145, 146). Moreover, PAF activity has been documented to be antagonized by PAF acetylhydrolase (PAF-AH), which catalyzes the deacetylation of PAF onto the sn-2 acetyl group of its glycerol backbone (147, 148). A previous study reported that PAF-AH deficiency was closely related to a higher incidence of asthma (149), and the administration of recombinant human PAF-AH could effectively block the late-phase pulmonary inflammation in allergic asthmatic mice (150).

#### TXA_2_

TXA**_2_** is mainly released by platelets upon stimulation and acts as an autocrine agonist (151, 152). TXA**_2_** release in the lung initiates airway smooth muscle contraction, causing airway hyperresponsiveness. Ozagrel hydrochloride (OKY-046), a TXA**_2_**-synthetase inhibitor, introduced to the Japanese market in 1992 to treat adult bronchial asthma, is widely acknowledged for its efficacy in improving lung function and reducing steroid use (41). Aspirin is an antiplatelet medicine that selectively inhibits platelet TXA**_2_** production at low doses (75 to 300 mg) (153); its role in treating asthma is further discussed in the next section of this review. TXA**_2_** has been established to exert its effects *via* the thromboxane prostanoid (TP) receptors (154, 155). A significant association has been reported between T924C polymorphism in the TP receptor gene and asthma in China, Japan, and Korea (41). Furthermore, the therapeutic effects of TP receptor antagonists such as GR32191, BAY u3405, and AA-2414 in asthma have been validated by Japanese research groups (41), which are not consistent with Western studies. A detailed description of this controversy can be found in another review (41).

## Antiplatelet Strategies in the Treatment of Allergic Asthma

Given the importance of platelets in many aspects of allergic asthma, various antiplatelet strategies have been applied in animal models or clinical trials to treat allergic asthma. The main antiplatelet targets include TXA_2_, ADP receptors, integrin αIIbβ3, thrombin receptors, *etc* (156) (**Table 1**).

**Table 1:** Antiplatelet strategies in treating allergic asthma.

Drug	Mechanism	Findings	References
Aspirin	Inhibitor of COX enzyme, inhibiting TXA_2_ generation	Aspirin reduces the relative risk of newly diagnosed adult-onset asthma	(157, 158)
		Aspirin does not yield beneficial effects in patients with mild-to-moderate persistent asthma	(159)
Ozagrel	Inhibitor of thromboxane synthetase, inhibiting TXA_2_ generation	Ozagrel decreases clinical symptom and reduces steroid use in asthma patients	(41, 160)
Clopidogrel	P2Y12R antagonist	Clopidogrel decreases airway hyperresponsiveness, eosinophilic infiltration, goblet cell hyperplasia, and type 2 cytokine production in OVA-induced allergic asthmatic mice	(161)
Prasugrel	P2Y12R antagonist	Prasugrel reduces airway hyperresponsiveness in patients with asthma	(162)
Ticagrelor	P2Y12R antagonist	Ticagrelor does not improve the pulmonary function of mild asthmatic patients	(163)
MRS2179 MRS2500	P2Y1R antagonist	MRS2179 and MRS2500 decrease pulmonary leukocyte recruitment in OVA-induced allergic asthmatic mice	(164)
Tirofiban	Integrin αIIbβ3 antagonist	Tirofiban decreases airway hyperresponsiveness, cytokine production, platelet-eosinophil aggregates, and eosinophilic infiltration	(72)

### TXA_2_ Synthesis Inhibitors

As mentioned, Aspirin and Ozagrel inhibit the TXA_2_ production (165). Aspirin is the most commonly used antiplatelet drug (166). It is well-established that the mechanism of aspirin involves acetylation of a serine residue on COX, leading to suppression of prostaglandins and TXA_2_ synthesis (156). Importantly, low doses of aspirin (75 ~ 300 mg) can selectively inhibit platelet TXA_2_ production and prevent cardiovascular disease (153). Existing evidence suggests that aspirin exerts a preventive effect on asthma: two large randomized, double-blind, placebo-controlled trials involving 22040 men and 37270 women revealed that aspirin could reduce the relative risk of newly diagnosed adult-onset asthma (22% lower in men and 10% lower in women) (157, 158). However, another study found that aspirin did not alleviate airway inflammation or pulmonary function in patients with mild-to-moderate persistent asthma (159). Ozagrel is widely used for asthma and stroke in Japan (167) and for stroke in China (165). On the contrary, it has not been approved by the Food and Drug Administration (FDA) or European Medicines Agency (EMA). It has been demonstrated that administration of Ozagrel can significantly decrease clinical symptom of asthma and reduce the dose of concomitant steroid therapy in Japanese clinical studies (160).

Nonetheless, it should be borne in mind that aspirin can induce a set of asthma-related symptoms in susceptible populations, referred to as aspirin-exacerbated respiratory disease or aspirin-induced asthma (168). Current evidence suggests that aspirin-induced asthma mainly results from dysregulation of arachidonic acid metabolism, leading to elevated levels of cysteinyl leukotrienes (CysLTs) and prostaglandin D_2_ (PGD_2_) and reduced levels of prostaglandin E_2_ (PGE_2_). It has been shown that elevated CysLTs and PGD_2_ can trigger bronchoconstriction, vascular leakage, recruitment of inflammatory cells, and mucus overproduction. On the other hand, PGE_2_ possesses anti-inflammatory properties and can induce bronchodilation (168). Surprisingly, aberrantly activated platelets have been documented in patients with aspirin-induced asthma rather than exhibiting an inhibition state as expected. Large numbers of platelet-adherent leukocytes have been reported in the peripheral blood and nasal polyp tissues of patients with aspirin-induced asthma. Indeed, low-dose aspirin (100 mg/d) does not reduce platelet-leukocyte aggregation. Moreover, adherent platelets contribute to the overproduction of CysLTs from leukocytes. Notwithstanding that it remains unclear whether platelets in patients with aspirin-induced asthma are intrinsic deficient, platelets have been demonstrated to act as effector cells in this disease (169). Therefore, aspirin does not inhibit platelet activity in this particular patient population; instead, it stimulates platelet activation and their interaction with leukocytes to promote airway inflammation.

### Purinergic Receptor Antagonism

Platelets express four types of purinergic receptors: P2Y1R, P2Y12R, P2X1R, and P2Y14R (170). As mentioned before, P2Y1R and P2Y12R represent ADP receptors, and P2X1R is an ATP receptor. P2Y14R is a receptor for UDP-glucose with no hemostatic function (171), while P2Y12R is a receptor for leukotriene E4 (LTE4), which is a powerful inducer of mucosal eosinophilia and airway hyperresponsiveness in asthma. It has been shown that platelet depletion could suppress the stimulatory effect of LTE4 on eosinophilia, goblet cell metaplasia, and IL-13 expression in OVA-induced allergic asthma mice (172). Furthermore, an association has been found between SNPs and haplotypes in *P2Y12* and lung function in a large family-based asthma cohort; house dust mite exposure resulted in significant gene-by-environment effects (173). P2Y12R is well-recognized as the target of commonly used purinergic receptor antagonists, such as clopidogrel, prasugrel, and ticagrelor (156). The therapeutic effects of clopidogrel, prasugrel, and ticagrelor in allergic asthma have been extensively investigated over the years. Substantial evidence suggests that clopidogrel decreases airway hyperresponsiveness, eosinophilic infiltration, goblet cell hyperplasia, and type 2 cytokine production in an OVA-induced allergic asthmatic mouse model (161). Clopidogrel has also been found to decrease platelet-eosinophil aggregates (174). Likewise, a proof-of-concept, placebo-controlled, randomized, cross-over study (26 asthmatic patients) demonstrated that prasugrel treatment (10 mg/day for 15 days) could reduce airway hyperresponsiveness in asthma patients (162). However, no significant improvement in pulmonary function was observed with ticagrelor (a single 450 mg dose, 180 mg 12 hours later, twice daily for 2 days, and once on day 4) in mild asthmatic patients (n=11) (163). The different effects of clopidogrel, prasugrel, and ticagrelor on allergic asthma may be attributed to their different anti-P2Y12 mechanisms since clopidogrel and prasugrel are irreversible, competitive, thienopyridine P2Y12 receptor antagonists, and ticagrelor is a direct-acting, non-competitive, reversible, cyclopentyl-triazolopyrimidine P2Y12 receptor antagonist (156).

Interestingly, P2Y1R activation also participates in the pathogenesis of allergic asthma. Studies have shown that treatment with P2Y1R antagonists, MRS2179 and MRS2500, could decrease pulmonary leukocyte recruitment in OVA-induced allergic asthmatic mice. The formation of the platelet-leukocyte complex is also largely dependent on platelet P2Y1R. An *in vitro* mechanistic study showed that the RhoA signaling downstream of platelet P2Y1R is required for platelet-dependent leukocyte chemotaxis (164). In contrast with other findings in the literature (161, 174), Amison and colleagues found that P2Y12R antagonists yielded no effect on pulmonary leukocyte recruitment (164), which may be attributed to the heterogeneity in experimental methods.

### Integrin αIIbβ3 Antagonism

Integrin αIIbβ3 is highly abundant on the platelet surface and plays key roles in platelet adhesion and aggregation, especially in the interaction between platelets, eosinophils (72), and neutrophils (26, 88). Current drugs targeting integrin αIIbβ3 include abciximab, eptifibatide, and tirofiban. However, these drugs have been associated with increased bleeding risk (156). Only tirofiban has been investigated in an OVA-induced allergic asthmatic mouse model to the best of our knowledge. Importantly, it has been found that tirofiban treatment could decrease airway hyperresponsiveness and cytokine production and attenuate platelet-eosinophil aggregates and subsequent eosinophilic infiltration (72).

### Thrombin Receptor Antagonism

The serine protease thrombin is well-recognized as one of the most potent platelet activators. Thrombin stimulates platelets by cleavage and activation of protease-activated receptors (PARs): PAR1 and PAR4. Moreover, vorapaxar is a common thrombin receptor antagonist, targeting PAR-1 (175). It has been suggested that thrombin generation is enhanced in blood serum and lungs of asthmatic patients (176, 177). Current reports suggest that thrombin promotes airway remodeling *via* PAR1 activation to induce TGFβ production in OVA-allergic rats (178). Moreover, in an OVA-induced allergic asthmatic mouse model, *PAR1* deficiency decreased airway inflammation and Th2 cytokine levels, whereas PAR1 agonists could exacerbate findings. It should be borne in mind that thrombin plays a dual role in allergic asthma: low-dose thrombin yields an inhibitory effect, while high-dose thrombin stimulates airway inflammation. This phenomenon may be attributed to thrombin preferentially binding to thrombomodulin on endothelial and airway epithelial cells to limit inflammation and binding to PAR1 to promote inflammation until thrombomodulin are saturated with thrombin (177). However, the effect of vorapaxar in allergic asthma remains primarily understudied, warranting further investigation.

In sum, the application of multiple antiplatelet therapies in treating allergic asthma has achieved great success in either animal models or clinical trials, such as clopidogrel, prasugrel, and tirofiban, which provides new options for the treatment of allergic asthma. However, antiplatelet drugs have not been applied to treat allergic asthma, which may be attributed to two aspects. First of all, there is a lack of long-term and large-scale clinical trials to confirm the treatment effect of antiplatelet drugs in allergic asthma. Most relevant studies have been carried out in small-scale clinical studies or animal models. For instance, clinical studies of prasugrel or ticagrelor in the treatment of allergic asthma only included 26 or 11 patients, and they were conducted in a short time (162, 163). Indeed, the increased risk of bleeding associated with current antiplatelet drugs is a prominent side effect (156) and a limitation for long-term clinical trials. Accordingly, retrospective and prospective analyses evaluating the treatment effect of antiplatelet drugs in patients with asthma and thrombotic disorders are of significant importance in the future. Besides, platelet exhibit different phenotypes in prothrombotic and proinflammatory conditions (21, 170). During hemostasis/thrombosis, aggregation is the most important function of platelets (179). In contrast, the most important function of platelets is to interact with other leukocytes in allergic asthma (21). Currently available antiplatelet drugs are designed to inhibit platelet aggregation, which may not significantly influence the interactions between platelets and leukocytes. Developing new therapies to block these interactions will be a breakthrough in the treatment of allergic asthma. P-selectin is a great target because, as mentioned earlier, it plays a crucial role in mediating platelet-eosinophil/neutrophil interactions. Crizanlizumab, an anti-P-selectin monoclonal antibody, has been approved by FDA in the USA to prevent vascular occlusion crises (VOCs) in sickle cell disease patients who express high levels of P-selectin (180). We hypothesize that crizanlizumab has huge prospects for application in treating allergic asthma in the future. In parallel, we also expect other new therapies to target interactions between platelets and leukocytes.

## Concluding Remarks

Allergic asthma is an intricate disease involving multiple types of immune cells. Accruing evidence from recently published studies suggests that platelets are activated and recruited to the lungs in allergic asthma, suggestive of their potential role in disease pathogenesis. Platelets mediate allergen sensitization by interacting with DCs and affect pulmonary recruitments and subsequent formation of EETs and NETs *via* interactions with eosinophils and neutrophils. In addition, it remains unclear whether platelets interact with mast cells and basophils in allergic asthma. The importance of mast cells and basophils in allergic asthma highlight the need for further studies to understand the underlying mechanisms. Furthermore, platelets serve as reservoirs of biochemical molecules, including various mediators that participate in inflammatory responses.

It should be noted that current antiplatelet drugs are based on the activation mechanisms of platelets in hemostasis/thrombosis, different from the current understanding of the role of platelets in allergic asthma. Unexpectedly, studies in asthmatic patients and animal models have confirmed that some antiplatelet drugs developed therein demonstrated beneficial effects in treating allergic asthma. We speculate that the potential mechanisms may be related to the similar activation characteristics of the platelet activation in the two pathological processes, such as P-selectin expression, integrin αIIbβ3 conformational change, secretion of mediators, regulated by these antiplatelet drugs. Indeed, these findings emphasize the need to develop new drugs to regulate proinflammatory functions by targeting platelet-DC/eosinophil/neutrophil interactions and the release of platelet-derived mediators to improve the treatment of allergic asthma.

## Author Contributions

All authors listed have made a substantial, direct, and intellectual contribution to the work, and approved it for publication.

## Funding

This work was supported by grants from the National Natural Science Foundation of China (82104164, 82003753, 82174140, 82174401, 81973870, 81804121), the Natural Science Foundation of Zhejiang Province (LQ19H080001, LY22H270003, LQ21H080002), Joint Funds of the Zhejiang Provincial Natural Science Foundation of China under Grant No. LBY22H270008, Traditional Chinese Medical Administration of Zhejiang Province (2022ZX005, 2022ZB119, 2021ZB090), Zhejiang Medical and Health Science and Technology Project (2021KY222), Research Project of Zhejiang Chinese Medical University (2021JKZDZC02, 2021JKZKTS036A, 2021JKJNTZ022B), National Undergraduate Innovation and Entrepreneurship Training Program (202110344005, 202110344025, S202110344007, 202010344004), General Research Project of Zhejiang Provincial Education Department “Special Project for the Reform of Cultivation Mode of Professional Degree Graduate Students in Higher Education Institutions” (Y202145932), Postgraduate Science Research Fund of Zhejiang Chinese Medical University (2021YKJ02, 2020YKJ07).

## Conflict of Interest

The authors declare that the research was conducted in the absence of any commercial or financial relationships that could be construed as a potential conflict of interest.

## Publisher’s Note

All claims expressed in this article are solely those of the authors and do not necessarily represent those of their affiliated organizations, or those of the publisher, the editors and the reviewers. Any product that may be evaluated in this article, or claim that may be made by its manufacturer, is not guaranteed or endorsed by the publisher.
